# Multivariate Analysis of the Determinants of the End-Product Quality of Manure-Based Composts and Vermicomposts Using Bayesian Network Modelling

**DOI:** 10.1371/journal.pone.0157884

**Published:** 2016-06-17

**Authors:** Julie Faverial, Denis Cornet, Jacky Paul, Jorge Sierra

**Affiliations:** 1 INRA, UR1321 ASTRO, Agrosystèmes Tropicaux, Petit-Bourg, Guadeloupe, France; 2 CIRAD, UMR AGAP, Montpellier, France; University of Illinois at Chicago, UNITED STATES

## Abstract

Previous studies indicated that the quality of tropical composts is poorer than that of composts produced in temperate regions. The aim of this study was to test the type of manure, the use of co-composting with green waste, and the stabilization method for their ability to improve compost quality in the tropics. We produced 68 composts and vermicomposts that were analysed for their C, lignin and NPK contents throughout the composting process. Bayesian networks were used to assess the mechanisms controlling compost quality. The concentration effect, for C and lignin, and the initial blend quality, for NPK content, were the main factors affecting compost quality. Cattle manure composts presented the highest C and lignin contents, and poultry litter composts exhibited the highest NPK content. Co-composting improved quality by enhancing the concentration effect, which reduced the impact of C and nutrient losses. Vermicomposting did not improve compost quality; co-composting without earthworms thus appears to be a suitable stabilization method under the conditions of this study because it produced high quality composts and is easier to implement.

## Introduction

The management of animal waste is a global issue of increasing importance as it has hazardous impacts on human health and the environment [1]. This is particularly important in the small island states of the Caribbean where the rises in population and food consumption during the past three decades have been accompanied by a shift in diet away from staples such as roots and tubers towards more livestock products [2].The livestock sector is thus evolving towards industrial farming methods that generate massive quantities of animal waste which is difficult to manage because of the limited availability of agricultural land for recycling [3]. In the French West Indies, only 10% of animal wastes are currently used on agricultural lands for spreading or composting, the remainder being improperly managed outside the framework of current regulations [4]. For local authorities, composting appears to be a sustainable solution to manage animal wastes from both environmental and agronomic point of views [5]. Indeed, the composting of animal wastes is an interesting practice regarding the restoration and improvement of weathered and infertile tropical soils, such as those in the Caribbean [6], and the replacement of chemical fertilizers in an export agriculture characterised by very intensive cropping systems [7]. Composting animal manures enables the export of nutrients from areas of high nutrient loading to soils which may be nutrient-deficient [8]. Many authors have highlighted the benefits of composting manure rather than other raw materials because it contributes to a significant supply of carbon (C) and nitrogen (N), while improving the soil organic matter (OM) content and physical properties such as density, porosity and water-holding capacity [9].

A key issue for composts produced under tropical conditions is that their quality, in terms of OM and nutrient content, is poorer than that of composts found in temperate regions. Previous studies reported that composts produced in the tropics presented lower C and nutrient levels than most composts produced in temperate regions [10, 11]. Because compost quality is markedly dependent on weather conditions during composting [12], a low OM content in tropical composts may be associated with high ambient temperatures that induce fast mass decomposition, even during the stabilization (or curing) phase of composting [10, 11]. As the procedure applied for composting also affects end product quality, several factors could be managed to improve compost quality in the tropics. For example, the use of raw materials rich in lignocellulosic compounds as a bulking agent, such as bagasse and wood chips, can ensure a high organic carbon content which increases the C/N ratio of the initial blend, leading to a reduction in OM and nutrient losses [13]. Vermicomposting is another practice that could improve end product quality because the greater mass reduction caused by worm activity during the stabilization phase induces a higher concentration of nutrients (i.e., the concentration effect) [14].

During composting, biological, physical and chemical processes operate simultaneously to affect compost quality, conferring a high degree of complexity on the composting process [15]. During the past decade, Bayesian networks have been widely applied to assessing such complex systems in agriculture; e.g., the impact of farmer practices on resilience to climate change [16] or analysis of the factors affecting crop yield variability [17]. Bayesian network modelling has the potential to reveal far more about key features of complex biological systems than the approaches commonly employed at present [18].

During this study, we assessed the underlying factors that affect the end quality of co-composts of manures and green wastes produced under a tropical climate. The aim was to evaluate three factors in terms of their impact on compost quality: i- the type of manure (cattle, goat or horse manure or poultry litter), ii- the value of co-composting with a bulking agent (e.g., composting of manures without or with green waste), and iii- the procedure applied during the stabilization phase (e.g., with or without earthworms). The study focused on the relationships between the chemical and biochemical characteristics of the raw materials, the mass, C and nutrient losses during the thermophilic and stabilization phases and the quality of the end product. The interactions between these variables were assessed using Bayesian networks.

## Materials and Methods

### Composting Procedure

The composting experiments were conducted at the Tropical Platform of Animal Experimentation of the French National Institute for Agricultural Research (INRA) in Guadeloupe (French West Indies; 16°20 N, 61°66 W, 114m a.s.1.). Specific permissions for this study were not required. The mean annual air temperature is 25.5°C at this site. We tested four different manures: cattle manure, goat manure, horse manure and poultry litter. The cattle and goat manures were collected from livestock living at the Platform cited above. Horse manure was collected from the stables of a local riding club, and poultry litter was collected on bed of wood chips from a local farmer. In both cases the owners gave us permission to collect their manures. The tropical grass *Dichantium aristatum* (Poir) C.E. Hubbard was used as green waste and was obtained from uneaten fodder left by goats raised in the same platform. The characteristics of raw materials are presented in Table 1.

**Table 1:** Some characteristics of the raw materials used during the study.

	pH	C/N	TC	LIG	N	P	K
Raw Material			% of dry matter				
Cattle manure	7.7^a^	21.0^b^	37.8^b^	16.7^a^	1.8^c^	0.5^b^	1.4^b^
Horse manure	8.0^a^	19.2^b^	32.7^c^	10.5^b^	1.7^c^	0.8^a^	1.4^b^
Goat manure	8.0^a^	20.2^b^	42.5^a^	11.5^b^	2.1^b^	0.8^a^	2.5^a^
Poultry litter	6.5^b^	10.2^c^	43.1^a^	6.5^c^	4.2^a^	0.9^a^	2.5^a^
Green waste	6.1^b^	27.5^a^	44.1^a^	9.6^b^	1.6^c^	0.3^b^	1.3^b^

TC, total C; LIG, lignin; N, total nitrogen; P, total phosphorus; K, total potassium.

For each property, values followed by different letters are significantly different at *P* <0.05.

Two treatments were applied to each animal manure: a compost of 100% manure and a co-compost of manure and green waste (50%/50% on a dry matter basis). A control treatment of 100% green waste was included in the experiment. There were therefore nine different manure/green waste treatments: four with pure manures, four co-composts and the control treatment. Each treatment had four replicates. For composting, piles of a pyramidal shape were prepared on an experimental scale (2 m high and 2 m basal diameter). The piles were placed in a shed to prevent excessive drainage and nutrient leaching due to heavy tropical rains. The total amount of dry matter in the piles was 150 kg. The temperatures inside the piles were measured at a depth of 0.4 m using thermocouples (Thermo Electric Co. Inc., Saddle Brook, NJ, USA) and recorded hourly by a data-logger (CR10X; Campbell Scientific, Shepshed, UK). The piles were turned manually to promote aeration and ensure that all the material was exposed to high temperatures during the thermophilic phase of composting. Turning was performed when the temperature inside the piles fell to 40°C. In this way, each pile was turned three times. The end of the thermophilic phase was determined when the temperatures inside the piles had remained lower than 32°C for five consecutive days.

At the end of the thermophilic phase, the remaining material in each pile was divided into two heaps of equal weight and placed in 80-L plastic containers. One container was used for vermistabilization and one container was used for stabilization without earthworms. For the vermicompost, we used a mix of *Eudrilus eugeniae* (Kinberg) and *Perionyx excavatus* (Perrier) earthworms collected from mounds of cattle manure and goat manure stored at the Platform. Earthworms were added at a rate of 1.5 kg per 100 kg of compost mass. The containers were kept in the shed and covered with a perforated plastic net. The procedure adopted for vermicomposting was based on the knowledge of local farmers. Covered containers were used to prevent the presence of local predators such as *Scolopendra subspinipes* Leach (scolopendra), *Eleutherodacty lusmartinicensis* Tschufi (Martinique robber frog), and *Bulbucus ibis* L. (cattle egret). The treatments without earthworms was also performed in containers to ensure the same environmental conditions for both stabilization methods. The temperatures inside the containers were measured as described above. It was not possible to vermicompost the pure poultry litter treatment because the high ammonia content in the composting mass at end of thermophilic phase affected worm survival. The composts and vermicomposts were watered when necessary to maintain the moisture content at 50–70%. Vermicomposting was considered to have been completed when the worms started leaving the composting mass. Taking account of the fact that temperature and moisture levels were suitable for worm activity throughout the composting process, we assumed that at this time food depletion was the key factor affecting worm survival within the composting mass. The treatments without earthworms were also halted at this time. A total of 68 end products were thus obtained; i.e., 36 composts and 32 vermicomposts, considering the four replicates for each end product.

Sampling of the composting mass from each pile was performed at the start and end of the thermophilic phase. For each container, sampling was performed at the end of the stabilization phase. For this, one composite sample (~3kg dry matter) of 15 sub-samples was collected from each pile (thermophilic phase) and from each container (stabilization phase) at a depth of 0.3–0.4 m. These samples were used for the analyses of pH, total C (TC), total N, total phosphorus (P), total potassium (K), pH and lignin content (LIG). The amount of dry matter in the remaining material was determined at the end of the thermophilic and stabilization phases and used to estimate mass, TC, LIG and nutrient losses at these time points.

### Chemical and Biochemical Analyses

Ammonium-N content was extracted from 20 g of fresh samples by shaking for 24 h with 100 mL of 0.5 M KCl and centrifuging. The measurement was made using a continuous flow colourimeter (AutoAnalyzer 3; Bran+Luebbe, Le Mans, France). Approximately 1 kg of each composite sample was lyophilised (Christ Alpha 1–4, Christ, Osterode, Germany) for chemical analyses. Sub-samples ground to <0.2mm were used to determine TC, N, P and K. Total C and N were determined using an elemental analyser (NC 2100Soil; CE Instruments, Milan, Italy). Phosphorus and K were determined after mineralization and digestion with HCl. Phosphorus was measured colorimetrically using a spectrophotometer (Cary 100; Varian Inc., Grenoble, France). Potassium was measured by atomic absorption spectrophotometry (AAFS 240; Varian Inc., Grenoble, France). Sub-samples ground to <2mm were used to determine the pH (compost: water 1: 10 w/v) and LIG content [19]. As proposed by some authors, in this study we considered the LIG content to be an index of compost stability [15]. All measurements were performed in duplicate.

### Estimate of Uncertainty of Mass and Nutrient Losses

Mass and nutrient losses were estimated as the difference between the compost or the nutrient mass at the beginning (m_ini_) and the end (m_end_) of each composting phase. The variance of that difference, VAR(m_ini_−m_end_), may be calculated as VAR(m_ini_) + VAR(m_end_)– 2 × COV(m_ini_, m_end_), where COV(m_ini_, m_end_) is the covariance between m_ini_ and m_end_. We calculated VAR(m_ini_-m_end_) to assess the uncertainty of mass and nutrient losses as estimated in this study. Calculations were performed using data of compost mass and nutrient content observed during the composting experiments.

### Explanatory Variables

We used three categories of explanatory variables describing the composting process, the compost properties and the losses during composting. The first group included co-composting/composting (referred to as co-composting in the networks), the length of the thermophilic phase (referred to as length) and the stabilization method (referred to as vermicomposting). The second group included the TC, N, LIG, P and K contents in the initial blend, at the end of the thermophilic phase and at the end of the stabilization phase, and the pH of the initial blend. The third group included losses of mass, TC, N, LIG, P and K during the thermophilic and stabilization phases. The C/N ratio was not included in the analysis to avoid redundancy with TC and N. All variables are continuous except for co-composting and vermicomposting, which are dummy variables. We developed four Bayesian networks. The first two were devoted to variables linked to OM (i.e., TC, LIG and N) during the thermophilic and stabilization phases, while the latter two focused on nutrient contents (i.e., N, P and K) during the thermophilic and stabilization phases. In this way, it was possible to link the networks with N.

### Exploratory Analyses

The effect of the main factors analysed during this study (i.e., manure, co-composting and vermicomposting) on losses and end product quality were assessed by ANOVA with a three way design and four replicates per treatment. The *P*-value was set to 0.05 for all the tests.

### Additive Bayesian Network Modelling

Bayesian networks were used for this study for several reasons. First, they are well suited to complex problems with numerous interacting variables: all statistical dependencies between all variables in the data are sought (e.g., relationship between the C content of the initial blend and C losses during composting). Interactions between numerous variables of interest during composting can therefore be addressed at the same time while allowing for highlighted compensatory effects (e.g., effect of mass loss on nutrient concentration). Moreover, Bayesian networks visually represent all relationships between variables in a system by means of connecting arcs. This graphical model facilitates the comprehension and interpretation of such a complex system. Secondly, although little preliminary knowledge of the structure of dependencies is available, it is still possible to use the data to learn about the evolution of the principal chemical components (e.g., C and nutrients) during the composting process. The aim of such a structure discovery analysis is then to perform a model search on the data to identify an optimum model structure (i.e. a model with the best goodness of fit to the observed data). Finally, Bayesian networks naturally focus on the relationships between actions (e.g., vermicomposting vs. composting) and natural processes (e.g., C loss vs. N loss). Thus, Bayesian networks can easily be supplemented with variables encoding managerial decisions that in turn affect the natural variables of the model [20].

All modelling was performed within the R software package using the abn library [21, 22]. The Bayesian network modelling approaches presented here are similar to those used by Lewis and McCormick [18]. Some dependence relationships between variables were banned from the structure discovery analysis in order to maintain a logical time frame (e.g., initial C content cannot be dependent on C losses, while the reverse might be true). An exact structure discovery approach was used to identify a globally optimum directed acyclic graph. A directed acyclic graph is a non-circular graphical structure consisting of nodes that are connected to each other with directed edges. The globally optimum directed acyclic graph is defined as the network with the maximum goodness of fit (i.e., the lowest log marginal likelihood) over all possible directed acyclic graph structures [23]. Once the globally optimum model had been identified then the next task was to check this model for over-fitting. To correct for such over-fitting, a parametric bootstrapping approach was used [24]. Ten thousand bootstrap datasets were generated and fitted using an identical exact model search. These simulations were generated using open source JAGS software [25] and the rjags library under R. We then removed all dependencies with insufficient statistical support to be considered as robust, which were not recovered in at least a majority (50%) of the bootstrap results. The result of the analyses was a statistically robust additive Bayesian network (referred to below as the final best directed acyclic graph).The parameters were interpreted as usual as posterior marginal mean effects for each covariate. The mean effects of the various variables in our study were estimated, together with their posterior 95% confidence intervals.

The results were represented visually by a directed acyclic graph comprising a set of nodes connected by directed links (arcs). Each node denotes a random variable and the arcs define a given factorisation of the joint probability distribution of all the variables. The usual notation involves squares for discrete nodes and circles for continuous nodes. In directed acyclic graphs, solid arcs indicate a positive relationship between two variables while dotted arcs denote a negative relationship. Each arc is labelled with the effect size indicator (i.e., the standardized marginal posterior median) which offers an indication of the strength of the dependency between the two variables linked by the arc.

Like any statistical model, this composting model was a simplified representation of reality and inevitably contained errors, including measurement errors that could lead to biased models [26]. Here, measurement error issues were minimized by using a large panel datasets (i.e., 68 end products and two composting phases). However, it is recommended that this model should not be used outside the range of experimental variations.

## Results

### Characteristics of the Raw Materials, Composts and Composting Process

The highest pH values were observed for horse and goat manures and the lowest pH were seen for poultry litter and green waste (Table 1). The highest C/N ratio was observed for green waste and the lowest value corresponded to poultry litter; the other manures presented similar C/N ratio. While the highest TC content was observed in poultry litter, goat manure and green waste, the highest LIG content corresponded to cattle manure. Poultry litter presented the lowest LIG content. The highest nutrient content was found in poultry litter, followed by goat manure.

For most composts, the pH rose by about 1.2 units during the thermophilic phase and then fell during the stabilization phase to reach values similar to those observed at the start of composting (data not shown). For composts of poultry litter, the pH rose to 10.4 during the thermophilic phase and then fell to 8.9 at the end of the stabilization phase. Ammonium-content in the initial blend was highest for composts of poultry litter and lowest for compost of cattle manure and green waste (Table 2). This content decreased during the thermophilic phase but it remained relatively high for poultry litter composts. At the end of the experiment, NH_4_-N was low and similar for all the composts.

**Table 2:** NH_4_-N content in the initial blend and at the end of the thermophilic and stabilization phases.

Compost	Initial blend	End thermophilic	End stabilization
	mg N-NH_4_ kg^-1^ dry matter		
Compost 100% CM	430^d^	221^c^	11^a^
Co-compost 50% CM /50% GW	558^d^	222^c^	13^a^
Compost 100% HM	2420^c^	149^c^	12^a^
Co-compost 50% HM / 50% GW	1554^c^	224^c^	12^a^
Compost 100% GM	2464^c^	261^c^	10^a^
Co-compost 50% GM /50% GW	1646^c^	286^c^	12^a^
Compost 100% PL	17226^a^	1279^a^	12^a^
Co-compost 50% PL /50% GW	8735^b^	471^b^	14^a^
Compost 100% GW	559^d^	284^c^	10^a^

CM, cattle manure; GW, green waste; HM, horse manure; GM, goat manure; PL, poultry litter.

Values correspond to the mean of the composting and vermicomposting treatments with four replicates (n = 8).

For each property, values followed by different letters are significantly different at *P* <0.05.

The total length of the entire composting process ranged from 138 d for composts produced using cattle manure to 205 d for composts produced from poultry litter. These differences were mainly due to variations in the length of the thermophilic phase; e.g., from 98 d to 148 d for the thermophilic phase and from 40 d to 57 d for the stabilization phase. The total length was about 15 d longer for co-composts than for the corresponding composts of pure manure. The maximum temperature reached during the thermophilic phase just before the first turning was 74°C; the maximum temperature fell by about 8°C after the second and third turnings. The average temperature inside the composting mass during the stabilization phase was 28°C.

The analysis of VAR(m_ini_−m_end_) indicated that uncertainty of mass losses, expressed as coefficient of variation, averaged 9% for the thermophilic phase and 13% for the stabilization phase. The values were slightly higher for nutrient losses (e.g. 12% and 15%, respectively) because the uncertainty associated to nutrient content. As vermicomposting had no a significant effect on losses of mass, C, LIG and nutrients, Table 3 presents the averaged losses of the compost and vermicompost treatments. All losses were greater during the thermophilic phase than during the stabilization phase. Total mass and TC losses were higher than other losses and averaged 50% for compost mass and 60% for TC. Mass and TC losses from composts produced using cattle, goat and horse manures were smaller than for the corresponding co-composts. The highest mass and TC losses throughout the composting process were observed for poultry litter and green waste composts. LIG losses during the entire composting process averaged 32% and were highest from co-composts of poultry litter (50%) and goat manure (39%). Total losses of N, P and K averaged 33%, 19% and 27%, respectively. Nutrient losses were higher from composts and co-composts of poultry litter. Composts of green waste and co-composts of poultry litter displayed the highest total K losses (45%).

**Table 3:** Losses of mass, carbon and nutrients during the thermophilic (ther) and stabilization (stab) phases.

Compost	Mass loss		TC loss		LIG loss		N loss		P loss		K loss	
	ther	stab	ther	stab	ther	stab	ther	stab	ther	stab	ther	stab
	% of the initial content											
Compost 100% CM	37^c^	3^c^	45^bc^	3^d^	25^a^	13^c^	13^cd^	1^c^	7^cd^	0^b^	20^b^	1^c^
Co-compost 50% CM / 50% GW	43^bc^	8^c^	52^b^	7^cd^	12^bc^	21^b^	16^cd^	2^c^	14^b^	4^b^	17^b^	9^bc^
Compost 100% HM	22^d^	3^c^	41^c^	10^c^	16^b^	6^c^	6^d^	9^c^	1^d^	5^b^	2^c^	7^c^
Co-compost 50% HM / 50% GW	45^bc^	7^c^	56^b^	13^c^	9^c^	21^b^	11^d^	8^c^	4^c^	7^b^	17^b^	9^bc^
Compost 100% GM	31^c^	1^c^	43^c^	4^d^	17^ab^	10^c^	20^c^	2^c^	12^bc^	0^b^	10^b^	2^c^
Co-compost 50% GM / 50% GW	61^a^	1^c^	68^a^	2^d^	31^a^	11^c^	39^b^	0^c^	24^a^	0^b^	35^a^	0^c^
Compost 100% PL	57^ab^	15^b^	64^ab^	24^b^	12^bc^	19^b^	68^a^	17^b^	6^cd^	19^a^	14^b^	14^b^
Co-compost 50% PL / 50% GW	49^b^	42^a^	59^ab^	42^a^	16^b^	41^a^	40^b^	41^a^	15^b^	28^a^	15^b^	35^a^
Compost 100% GW	54^ab^	20^b^	63^ab^	21^b^	6^c^	23^b^	16^cd^	21^b^	8^c^	23^a^	35^a^	16^b^

CM, cattle manure; GW, green waste; HM, horse manure; GM, goat manure; PL, poultry litter.

TC, total carbon; LIG, lignin; N, total nitrogen; P, total phosphorus; K, total potassium.

Values represent the losses of each element relative to its level at the start of the respective phase, and correspond to the mean values of the composting and vermicomposting treatments with four replicates (n = 8).

For each property, values followed by different letters are significantly different at *P* <0.05.

In the same way as for losses, vermicomposting had no a significant effect on the final compost properties (Table 4). On the contrary, the effect of manure type was significant relative to all compost properties. The lowest TC contents were observed in composts of horse manure, while the other composts presented similar TC contents (Table 5). The LIG content was highest in green waste composts, followed by composts of cattle manure. Low LIG levels were found in composts of horse manure and poultry litter. The final C/N ratio averaged 11 for most composts, while it was 8 for poultry litter composts. The ratio was slightly higher for co-composts than for the 100% manure treatments, but the differences were no significant (Table 5). The highest nutrient content was seen in composts of poultry litter, followed by composts of goat manure. The effect of co-composting was only significant with respect to TC, LIG and P (Table 4). Thus co-composts displayed higher TC and LIG contents and lower P contents (Table 5).

**Table 4:** Results of ANOVA for the effect of the three factors tested in this study on the quality of the end products.

Factor	df	TC	LIG	N	P	K
		*F*-value				
Manure	3	14.8 *	69.4 *	137.2 *	299.6 *	252.8 *
Co-composting	1	23.3 *	6.1 *	3.3 ns	72.5 *	3.8 ns
Stabilization method	1	3.4 ns	0.3 ns	0.3 ns	1.0 ns	0.2 ns

TC, total carbon; LIG, lignin; N, total nitrogen; P, total phosphorus; K, total potassium.

Manure: cattle manure, horse manure, goat manure and poultry litter; Co-composting: with or without green wastes; Stabilization method: with or without earthworms.

*, the effect of the factor was significant at *P*<0.05; ns, not significant.

**Table 5:** Chemical properties of the initial blend (ini) and of composts at the end of the experiment (end).

Compost	C/N		TC		LIG		N		P		K	
	ini	end	ini	end	ini	end	ini	end	ini	end	ini	end
			% of dry matter									
Compost 100% CM	18.9^bc^	10.8^bc^	36.6^b^	32.1^a^	18.9^a^	19.9^a^	1.94^c^	2.96^b^	0.6^c^	0.9^d^	1.4^b^	2.0^b^
Co-compost 50% CM / 50% GW	22.6^b^	11.5^b^	39.1^b^	33.1^a^	14.6^ab^	21.0^a^	1.73^c^	2.87^b^	0.4^d^	0.8^d^	1.5^b^	2.3^b^
Compost 100% HM	15.9^c^	9.5^c^	29.5^c^	20.4^b^	11.1^bc^	11.6^c^	1.86^c^	2.14^c^	1.0^a^	1.3^c^	1.3^b^	1.5^b^
Co-compost 50% HM / 50% GW	21.8^b^	9.7^c^	36.2^b^	26.1^b^	10.0^bc^	14.4^b^	1.66^c^	2.69^c^	0.7^bc^	1.3^c^	1.6^b^	2.6^b^
Compost 100% GM	19.5^bc^	10.1^bc^	42.1^a^	32.8^a^	12.3^b^	15.4^b^	2.13^b^	3.26^b^	1.0^a^	1.7^b^	2.7^a^	4.6^a^
Co-compost 50% GM / 50% GW	24.1^b^	9.4^c^	43.0^a^	34.0^a^	10.7^b^	16.9^b^	1.78^b^	3.06^b^	0.7^b^	1.5^bc^	2.4^a^	4.4^a^
Compost 100% PL	8.3^d^	7.6^d^	43.6^a^	32.5^a^	5.20^d^	11.9^c^	5.24^a^	4.29^a^	1.1^a^	2.3^a^	2.9^a^	5.9^a^
Co-compost 50% PL / 50% GW	12.8^c^	8.8^cd^	42.6^a^	34.6^a^	7.85^d^	14.2^b^	3.32^a^	3.95^a^	0.7^b^	1.4^c^	2.2^a^	4.3^a^
Compost 100% GW	28.7^a^	12.4^a^	43.1^a^	35.9^a^	9.80^c^	22.5^a^	1.50^d^	2.90^b^	0.3^e^	0.6^d^	1.7^b^	2.6^b^

CM, cattle manure; GW, green waste; HM, horse manure; GM, goat manure; PL, poultry litter.

TC, total carbon; LIG, lignin; N, total nitrogen; P, total phosphorus; K, total potassium.

Values for the composts represent the mean values of the composting and vermicomposting treatments with four replicates (n = 8).

For each property, values followed by different letters are significantly different at *P* <0.05.

### Bayesian Network Modelling of the Composting Process

**Fig 1:**
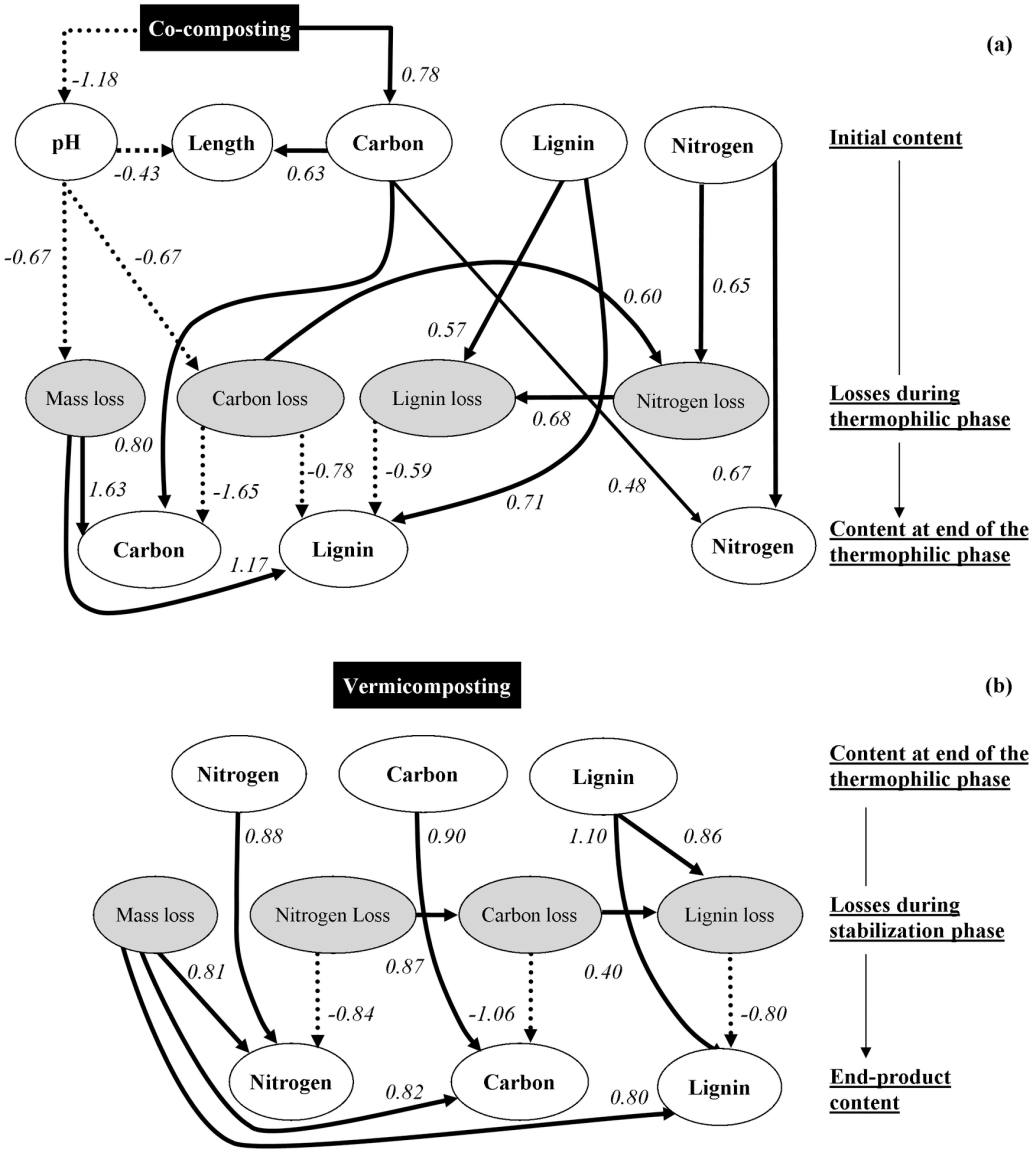
Directed acyclic graph of the final best multivariate regression model of variables describing changes in total carbon, lignin and nitrogen contents during the (a) thermophilic and (b) stabilization phases (Although shown, vermicomposting was not linked with any variables). The analysis was performed using an exact search additive Bayesian model. Solid and dotted arcs represent positive and negative links between variables, respectively. Arcs are labelled with the effect size (i.e., standardized median marginal posterior density).

**Fig 2:**
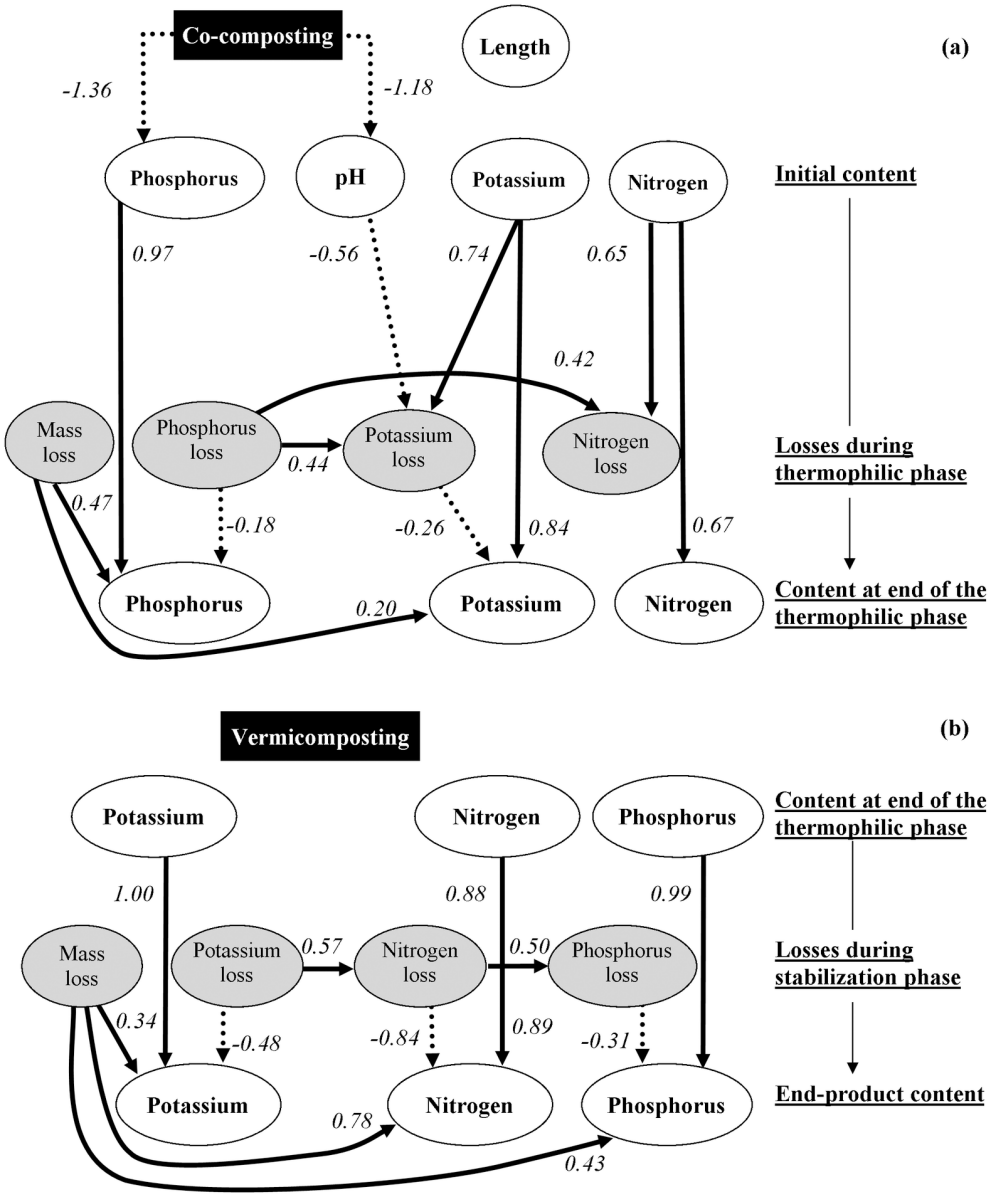
Directed acyclic graph of the final best multivariate regression model of variables describing changes in phosphorus, potassium and nitrogen contents during the (a) thermophilic and (b) stabilization phases (Although shown, vermicomposting was not linked with any variables). The analysis was performed using an exact search additive Bayesian model. Solid and dotted arcs represent positive and negative links between variables, respectively. Arcs are labelled with the effect size (i.e., standardized median marginal posterior density).

Figs 1 and 2 present the Bayesian networks for variables linked to OM and nutrient contents, respectively. For both the thermophilic and stabilization phases, and both OM and nutrients, the final properties mainly depended on the initial status and on the concentration effect induced by mass losses (Figs 1 and 2). Although the effect of losses was also important to determine the final content of OM and nutrients, this was also controlled by their initial status. In most cases, the losses were linked to each other. Mass losses positively affected the final contents, except for N during the thermophilic phase (Fig 1a). In fact, N in the thermophilic phase displayed some differences from the other properties insofar as its final content was not linked to N losses but was directly affected by the initial C content. The results obtained from the Bayesian networks confirmed that vermicomposting did not affect either losses or the final levels of the properties analysed (Figs 1b and 2b). The length of the thermophilic phase was linked to the initial C content and pH but had no interactions with the network of other compost variables. Co-composting positively affected the length of the thermophilic phase via the initial TC content and pH (Fig 1a). Co-composting also positively affected mass, TC (Fig 1a) and K losses (Fig 2a) via the pH, and negatively affected the initial P content (Fig 2a).

## Discussion

The Bayesian networks developed during this study constituted a helpful tool to identify the principal factors and processes affecting the end quality of composts. The major finding of this study was that the importance of these factors and processes differed for the two criterion used to define compost quality; e.g., amount and stability of OM and nutrient content. In this section we discuss separately both these indicators of compost quality.

### Organic Matter Content and Stability

The C/N ratio of the end products was within the range of values commonly reported for mature composts [8, 15]. As mentioned above, the C/N ratio was not included in the Bayesian networks to avoid redundancy with TC and N. In both composting phases, the initial content of TC, LIG and N affected their final content in two ways: directly and indirectly via their links with the losses of these elements. The LIG content is positively correlated with compost stability [15, 27]. It can therefore be concluded that the initial quality of the blend was one of the main factors affecting the amount and stability of OM in the end product. Moreover, losses of TC, LIG and N were much greater during the thermophilic phase than during stabilization, which implies that the thermophilic phase was the main period determining the quality of the end product under the conditions of this study. Also, the uncertainty of the estimated losses was lower for the thermophilic phase, which confirms the importance of that phase to control compost quality. The length of this phase was not linked with either losses or the final contents of the chemical elements, and subsequently did not affect the quality of the end products. However, this variable has a practical interest because raw materials with high biodegradability and a high TC content, such as poultry litter, presented a very long thermophilic phase, which could be a constraint for composting in industrial units [28].

The important mass and TC losses observed during the thermophilic phase is a well documented process, due to the rapid mineralization of organic substrates at the start of the composting process [15]. These losses were highest for poultry litter and green wastes composts with the lowest initial LIG content and then low OM stability. For the same reason, mass and TC losses were generally higher in co-composts with green waste than in the corresponding pure manure composts. LIG losses were relatively high during the thermophilic phase (e.g., an average of 16%). Tuomela et al. [29] reported that low N availability is often a prerequisite for lignin degradation, which might explain the positive link between N and lignin losses observed during the thermophilic phase. Nitrogen losses during the thermophilic phase were likely induced by leaching with excess water arising from OM decomposition and flowing through the composting mass by gravitational flux [30]. Losses by N volatilization could not be discounted in the horse and goat manure composts with a high initial pH and NH_4_-N content [15], despite the fact that the Bayesian network indicated that initial pH was not linked to N losses. Nitrogen volatilization could be more evident in composts produced from poultry litter where NH_4_-N content was extremely high (Table 2). These composts displayed the highest N losses which continued even during the stabilization phase; this was likely associated with the rise in pH that occurred during the composting process, and the relatively high NH_4_-N content observed at the end of the thermophilic phase. This affected earthworm survival, and vermicomposting could not be performed in the case of the 100% poultry litter treatment. The lack of correlation between the length of the thermophilic phase and N losses suggests that N leaching and volatilization were controlled by compost properties such as the total and the mineral N content, pH and water content rather that by the duration of the composting process. This implies that the management of the compost properties would be more important to decrease N losses than reducing the length of the thermophilic phase.

Except for N in the poultry litter composts, losses during the stabilization phase were relatively small. This result disagreed with a previous study carried out in Guadeloupe on cattle manure/green waste composts [11]. The losses reported by these authors regarding the stabilization phase accounted for approximately 30% of the losses observed during the entire composting process. In the present study, this value was approximately 10%. This could perhaps be attributed to differences in temperature inside the compost mass during the stabilization phase (i.e., 32°C in the study by Sierra et al. [11] and 28°C during the present study), which could affect OM mineralization, the release of mineral N and N leaching in that phase. As a consequence of this, the final TC content observed in the cattle manure/green waste composts studied here were 35% higher than those reported by these authors. In view of the fact that both studies were carried out in the same season, these results might reflect the impact of inter-year variability in temperatures during the stabilization phase on compost quality.

The second factor affecting the final contents of TC, LIG and N was mass loss via a concentration effect. This effect was stronger than that of the initial content (Fig 1a). For example, because LIG and N losses were smaller than the mass loss (Table 3), their content increased during the composting process (Table 5). The contrary was observed for TC, where the losses were higher than the mass loss. The concentration effect was very important regarding the LIG content in green waste compost, which displayed a low initial LIG content and the highest content in the end product (Table 5). However, the Bayesian network indicated that loss of mass during the thermophilic phase was not linked to the final N content (Fig 1a). We think this was due to the 100%-poultry litter based compost, which was the only compost where N losses were much higher than mass loss (Table 3). For the other composts, there was indeed a concentration effect and the final N content was always higher that the initial N content (Table 5). A concentration effect on final N content had already been reported for composts of urban wastes produced under tropical conditions [10]. In our study, a partial offset between the positive concentration effect and the negative effect of N losses could explain the lack of correlation between N losses and N content at the end of the thermophilic phase (Fig 1a).

The negative effect of pH on mass loss was surprising because pH was included in the network in order to test its impact on N losses by volatilization. It seems that such a negative relationship was induced by composts produced using poultry litter and green waste, which presented relatively low pH levels and at the same time a low LIG content that increased the losses of mass and TC. Therefore, this correlation could not reflect a mechanistic relationship between the two variables, but an indirect effect of the biodegradability of OM. It is interesting to note that the standardized effect size of mass loss on the final TC and LIG contents was higher during the thermophilic phase than in the stabilization phase, which confirms the greater importance of the thermophilic phase in determining the end product quality in terms of the OM quantity and quality.

Co-composting played a major role by controlling the two main factors affecting the final amount and stability of compost OM. First, co-composting directly affected the TC content of the initial blend because green waste contained higher TC levels than most manures. Second, it indirectly affected mass loss, and then the concentration effect, because green waste decomposed more rapidly than manures because of its lower LIG content [15]. Moreover, the impact of co-composting on the initial TC content also affected the final N content, which suggests that C was useful to retain N in the composting mass. This agrees with the proposal of several authors who reported that the use of a bulking agent might reduce N losses by contributing to the reorganisation of mineralized N [31].The slightly greater length of the thermophilic phase observed for co-composts seems a minor constraint when compared with the benefits procured by co-composting.

The stabilization method had no impact on the final amount and stability of OM. Positive [11, 31, 32] and negative [11, 33] impacts of vermicomposting on the quality of the end product have been reported previously. In general, the positive effects were associated with greater OM decomposition and a higher concentration effect, and the negative effects were induced by earthworm burrowing and mixing which enhanced N losses. In the study carried out in Guadeloupe cited above, vermicomposts displayed a lower OM content and greater stability than those observed for the composts produced without earthworms [11]. Taking into account that the procedure used in that study for the stabilization phase was the same than that applied here (i.e., closed plastic containers), we reject the conclusion that the “no impact” of vermicomposting observed in the present study was due to a methodological artefact. The comparison of both studies indicates that the effect of the stabilization method may vary markedly, even at the same composting site. This in agreement with the results reported by Neher et al. [34] who found that the stabilization method (e.g., windrow vs. vermicomposting) affected the structure of the microbial communities in the composting mass, which was controlled by the temperature regime. As discussed above, differences between our study and that of Sierra et al. [11] about the effect of vermicomposting might be associated to the inter-year variability in temperature during the stabilization phase.

From these results, it appears that co-composts of cattle manure and green waste may offer a high degree of OM stability and adequate levels of TC and N. It would constitute the best organic amendment when the aim is to maintain the soil OM content and recycle animal and plant wastes together. Co-composts of poultry litter, and secondarily of goat manure, offer a high N content and could be used as organic fertilizer. One constraint attached to these composts is their long thermophilic phase. For poultry litter, unpleasant odours during composting may be a serious constraint when composting in industrial units.

### Nutrient Content

Although the general pattern of the Bayesian network for nutrient content was similar to that obtained for OM variables, the weighting of the factors affecting compost quality was different. For example, the link with the final nutrient content was stronger for the initial contents than for mass losses; that is, the concentration effect was lower for nutrient than for OM content. This difference was most likely due to the fact that nutrient losses were smaller than those of TC and LIG. Potassium losses were likely associated with the leaching of soluble salts due to the weak retention exercised by OM on this element [11, 35]. Relatively low P losses in both composting phases might be attributed to the high Ca content in all the raw materials (e.g., >5% on dry matter basis; data not shown), which could promote the precipitation of P released during OM decomposition and prevent the leaching of this nutrient [36]. Unlike those seen for OM, relatively high losses were observed during the stabilization phase (e.g., 33% for K and 42% for P of total losses), indicating a partial shift between OM decomposition during the thermophilic phase and K and P leaching during the stabilization phase. Similar results have been reported by other studies carried out under tropical conditions, and were attributed to the gradual accumulation of soluble compounds and their transfer in water towards the bottom of the compost pile [10, 35]. The effect of co-composting also differed with respect to OM. Co-composting negatively affected both the initial and the final P content, which agreed with the results reported by Leconte et al. [35] for tropical composts. In addition, co-composting did not directly affect the final K content (Table 4), and its effect varied markedly between the different manures (Table 5), indicating an offset between K losses and the concentration effect.

As the effect of co-composting on the final P and K contents was small, and that of vermicomposting was nil, the type of manure was the principal factor determining compost quality in terms of its nutrient content (Table 4). Thus raw materials rich in nutrients, such as poultry litter and goat manure, produced composts with a higher nutrient content. This confirms that these raw materials could be used to produce organic fertilizers rich in N, P and K.

## Conclusions

Bayesian network modelling was found to be useful in assessing the impact of the raw materials and the procedure of composting on the end product quality. The concentration effect and the quality of the raw materials were the principal factors affecting the OM and nutrient contents and OM stability in the final composts. The co-composting of manures and green waste attenuated the effects of the tropical climate on OM losses by enhancing the concentration effect. The thermophilic phase presented high OM, lignin and N losses, which could be reduced by increasing the surface/volume ratio of the composting mass in order to enhance heat transfer. Moreover, the results indicated that the management of compost properties during the thermophilic phase would be more important to decrease N losses than reducing the length of that phase. Vermicomposting did not improve compost quality; composting without earthworms in the stabilization phase therefore appears to be an appropriate method because it produced high quality composts and is easier to achieve.

## Supporting Information

S1 File Manuscript Database. Physical, chemical and biochemical compost raw data which were analyzed for the current manuscript, are available and arranged by phases (initial and end of thermophilic and stabilization phases).
(XLS)
